# *Atractylodes macrocephala* Koidz. Polysaccharide Alleviates Chemotherapy-Induced Depression-Like Behaviors Through the Gut–Brain Axis

**DOI:** 10.3390/ijms262010189

**Published:** 2025-10-20

**Authors:** Zheng Liang, Yihan Yuan, July Chen Liang, Yingchao Wu, Jiaqi Cui, Haihong Gu, Dajin Pi, Zhongjia Yi, Shuyao Zhou

**Affiliations:** 1 The Second Clinical Medical College, Guangzhou University of Chinese Medicine, Guangzhou 510405, China; liangzheng@stu2021.jnu.edu.cn (Z.L.); 20242610555@stu.gzucm.edu.cn (J.C.L.); yc1996@stu2020.jnu.edu.cn (Y.W.); pdj9642@stu2019.jnu.edu.cn (D.P.); 2 College of Traditional Chinese Medicine, Jinan University, Guangzhou 510632, China; yuanyihan@stu2021.jnu.edu.cn (Y.Y.); kiki8@stu2023.jnu.edu.cn (J.C.); 3 The Graduate School, Zhejiang Chinese Medical University, Hangzhou 310053, China; 20225001051@zcmu.edu.cn; 4 College of Forestry and Landscape Architecture, South China Agricultural University, Guangzhou 510642, China

**Keywords:** *Atractylodes macrocephala* Koidz. polysaccharide, ferroptosis, depression, intestinal flora, hippocampus

## Abstract

This study explored the potential therapeutic effect and possible mechanism of *Atractylodes macrocephala* Koidz. Polysaccharide (AP) on pirarubicin chemotherapy-induced depression (CID) in breast cancer mice. This study utilized a variety of techniques to explore the potential of AP in mitigating behavioral abnormalities and elucidate the role of gut microbiota regulation in its therapeutic effects on chemotherapy in breast cancer mice. These included a chemotherapy mouse model, behavioral assessments, histological analysis using hematoxylin and eosin staining, ultrastructural examination, enzyme-linked immunosorbent assays, 16S rDNA sequencing, metabolomic profiling, Western blot analysis, and a pseudo-germ-free animal model. Oral administration of AP significantly improved depression-like behaviors in breast cancer chemotherapy mice while also reducing neuronal damage and inflammation in the hippocampus. AP prevented ferroptosis of intestinal tissues caused by chemotherapy and had a repairing effect on the intestinal barrier damage of chemotherapy-induced mice. Additionally, AP enhanced gut microbiota composition and altered intestinal metabolites in chemotherapy-treated mice. It notably decreased the abundance of certain microbes, such as *Bacteroidaceae*, *Lachnospiraceae*, *Oscillospiraceae*, and *Clostridium*, while significantly increasing the abundance of *Alistipes*. Moreover, AP efficiently modulated intestinal metabolites, including glycocholic acid, L-Phenylalanine, and palmitoylcarnitine. More importantly, depletion of gut microbiota through antibiotics diminished the effectiveness of AP. Our results suggest that AP alleviates depression-like behaviors in chemotherapy-treated mice by regulating the gut microbiota and microbial metabolism, as well as suppressing ferroptosis in intestinal tissues.

## 1. Introduction

According to data released by the American Cancer Society in 2024, breast cancer ranked first among all newly diagnosed cancers in women, accounting for 32% of female cancer cases [1]. Despite the evolution of systemic therapies for breast cancer from traditional chemotherapy to contemporary modalities such as endocrine therapy, targeted therapies, and immunotherapy, chemotherapy retains an indispensable position in clinical management [2]. Recent paradigm shifts in cancer therapeutics have heightened clinical attention to treatment-emergent adverse events. Among these, chemotherapy-induced depressive-like behavior represents a frequently observed complication in the systemic management of breast cancer [3]. Chemotherapy-induced depression (CID) affects a considerable number of cancer patients, substantially compromising global quality of life and diminishing treatment adherence [3]. The pathogenesis of CID primarily involves neurotoxic effects of chemotherapeutic agents on emotional regulation neural circuits, triggering neuroinflammatory infiltration and neuronal injury that collectively culminate in functional impairments manifesting as depressive-like behaviors [4,5]. Notably, most chemotherapeutic agents cannot cross the blood–brain barrier (BBB) to exert direct neurotoxic effects [5,6]. Consequently, these drugs likely mediate their neuropathological consequences through indirect pathways that ultimately culminate in neuronal injury and subsequent depressive-like behaviors.

Recent clinical studies suggest that the gut–brain axis may play a pivotal role in mediating CID [7]. The adverse effects of chemotherapy primarily stem from the non-selective nature of chemotherapeutic agents, which cause indiscriminate damage to healthy cells, particularly those exhibiting high proliferative activity [8]. Chemotherapeutic agents exemplified by pirarubicin, employed in breast cancer treatment, induce compromise of intestinal barrier integrity due to their cytotoxic actions [9,10]. This subsequently triggers systemic inflammation, including that in the central nervous system, ultimately leading to hippocampal nerve damage and induction of depressive-like behaviors [11]. Although the pathogenic role of gut-derived metabolites and inflammatory cascades in CID is increasingly recognized [12,13], effective interventions capable of comprehensively ameliorating these multisystem disturbances without compromising chemotherapeutic efficacy remain relatively scarce. However, current management strategies, including conventional antidepressant agents, frequently have poor efficacy and carry inherent risks of adverse effects [14,15,16].

Natural compounds possessing neuroprotective, anti-inflammatory, and gut-protective properties represent a promising therapeutic approach for neuro-related diseases such as CID and Alzheimer’s disease [5,12,17,18]. Among these, plant polysaccharides have garnered significant attention due to their possession of multiple bioactive properties, notably immunomodulatory and gut-protective effects [12,19,20,21]. Previous research demonstrated that polysaccharide extracts derived from *Atractylodes lancea* (Thunb.) DC preserve intestinal barrier integrity against drug-induced damage through the modulation of intestinal flora [12]. Regrettably, in previous research, there are no reports observing that *Atractylodes lancea* (Thunb.) DC polysaccharide has neuroprotective effects or the ability to prevent CID. Guided by traditional Chinese medicine principles and building upon preceding findings [22,23], we believe that *Atractylodes macrocephala* Koidz. polysaccharide (AP, also written as PAMK in some studies) may confer dual protective benefits, namely preserving intestinal barrier integrity against chemotherapy-induced damage while potentially mitigating CID through the gut–brain axis. Preliminary evidence suggests that AP may promote gastrointestinal health and exert neuroprotective effects [22,24]. However, their therapeutic potential against CID remains notably underexplored in preclinical settings. As discussed earlier, elucidating the underlying mechanisms and therapeutic efficacy of AP in CID would not only advance our understanding of gut–brain axis pathology but also hold significant translational promise for developing adjuvant neuroprotective strategies in oncology.

This study employed 16S rDNA sequencing, gut metabolomics, and a pseudo-germ-free animal model to investigate the pathological mechanisms underlying pirarubicin chemotherapy-induced depressive-like behaviors, demonstrating that AP attenuates CID through the gut–brain axis.

## 2. Results

### 2.1. Structural Analysis of AP

**Figure 1:**
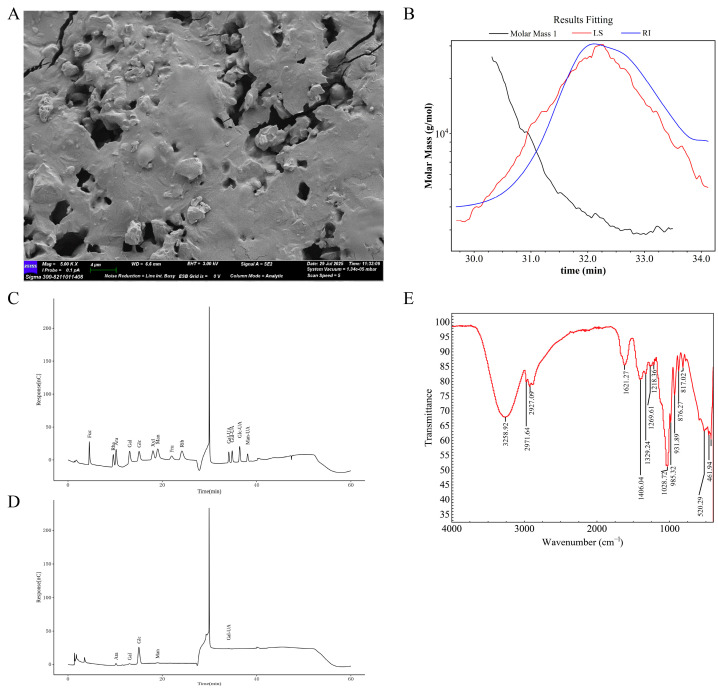
Physical and chemical characterization of AP. (**A**) Scanning electron micrographs. (**B**) Molecular weight distribution. (**C**,**D**) Monosaccharide composition analyses. (**E**) FT-IR spectrum.

The microscopic morphology of AP was characterized via SEM, and the results show that the surface of AP exhibited irregular sheet-like structures of different sizes, uneven overall morphology, and rough surfaces (Figure 1A), which may be related to its extraction methods, monosaccharide compositions, branching structures, bond strengths, and other factors. Subsequently, we determined the molecular weight of AP via GPC-RI-MALS, and the results show that the weight-average molecular weight (Mw) of AP was 4.254 kDa, number-average molar mass (Mn) was 3.642 kDa, and polydispersity index (Mw/Mn) of AP was 1.168 (Figure 1B). The monosaccharide composition of the polysaccharide contributes greatly to the functional characteristics and biological activity of the polysaccharide. Therefore, we then analysis the monosaccharide composition of AP via HPLC. Figure 1C shows the HPLC curve of the mixed standard and all standards were very well separated. We then compared the retention time of AP with that of standard monosaccharides and found that AP was mainly composed of arabinose, galactose, glucose, mannose, and galacturonic acid (Figure 1D). FT-IR analysis can reveal different functional groups and facilitate the analysis of different characteristic structures. FT-IR spectra of AP were recorded at the absorbance mode from 4000 to 400 cm^−1^ at a resolution of 4 cm^−1^ with 128 co-added scans, and the results are shown in Figure 1E. The infrared spectrum of the AP shows characteristic absorption bands in the 3600–3200 cm^−1^ range, attributed to the O-H stretching vibration of hydroxyl groups, which is a typical feature of carbohydrates. Specifically, the peak at 3258.92 cm^−1^ corresponds to the O-H stretching vibration, while the absorption at 2927.09 cm^−1^ is linked to the C-H stretching vibration, and the peak at 1028.72 cm^−1^ is associated with the C-O stretching vibration. These findings confirm the presence of carbohydrate-related functional groups in the AP sample.

### 2.2. AP Alleviates Depression-like Behaviors Caused by Chemotherapy

**Figure 2:**
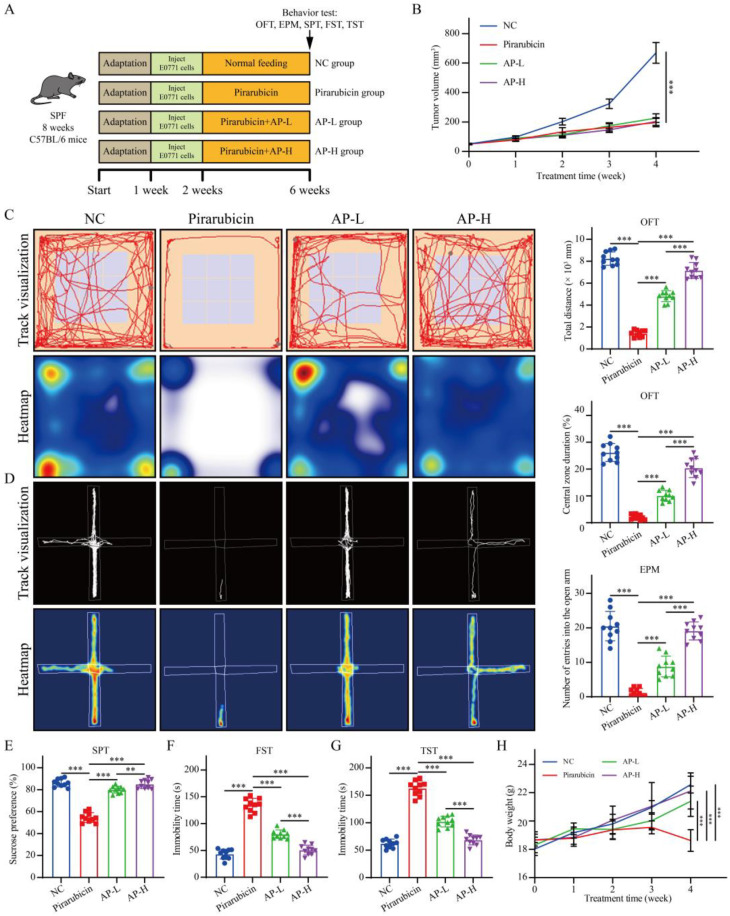
AP significantly mitigated depression-like behaviors in chemotherapy-treated mice. (**A**) Overview of study design and key experimental timeline. (**B**) Tumor size was measured every week. (**C**) Track map, heatmap, total distance, and central zone duration in OFT. (**D**) Track map, heatmap, and number of entries into the open arm in EPM. (**E**) Sucrose preference test (SPT). (**F**) Immobility time in FST. (**G**) Immobility duration in TST. (**H**) Body weight was measured every week. Data are presented as mean ± SD; comparison between the two groups: ** *p* < 0.01, *** *p* < 0.001; *n* = 10.

Figure 2A shows the experimental flowchart. Tumor observation results demonstrate that AP exerted no significant impact on the anti-tumor efficacy of pirarubicin against breast cancer (Figure 2B). Behavioral assessments, including the OFT, EPM, SPT, TST, and FST, were conducted to evaluate the impact of AP on depression-like behaviors in chemotherapy-treated mice. The total distance moved, and the time spent in the central zone during the OFT was significantly lower in the pirarubicin group compared to the NC group. In contrast, the AP-L and AP-H groups exhibited an opposite trend in these OFT parameters, with statistically significant differences compared to the pirarubicin group (Figure 2C). The mice exposed to pirarubicin chemotherapy showed a significant decrease in the number of entries in the open arm of the EPM compared to the NC group, and this reduction trend could be significantly reversed with AP (Figure 2D). SPT results demonstrate that the sucrose preference rate in the pirarubicin group was markedly lower than in the NC group. In contrast, mice in the AP-L and AP-H groups exhibited significantly increased sucrose preference compared with the pirarubicin group (Figure 2E). FST and TST results reveal that mice in the pirarubicin group had significantly longer immobility times than those in the NC group. In contrast, immobility times were markedly reduced in both the AP-L and AP-H groups compared with the pirarubicin group (Figure 2F,G). Furthermore, behavioral analyses indicated that the amelioration of CID with AP exhibited dose dependency (Figure 2C–G). Compared with the NC group, pirarubicin administration significantly decreased body weight in mice, which was subsequently ameliorated via AP treatment (Figure 2H).

### 2.3. AP Prevents Hippocampal Damage Caused by Chemotherapy

**Figure 3:**
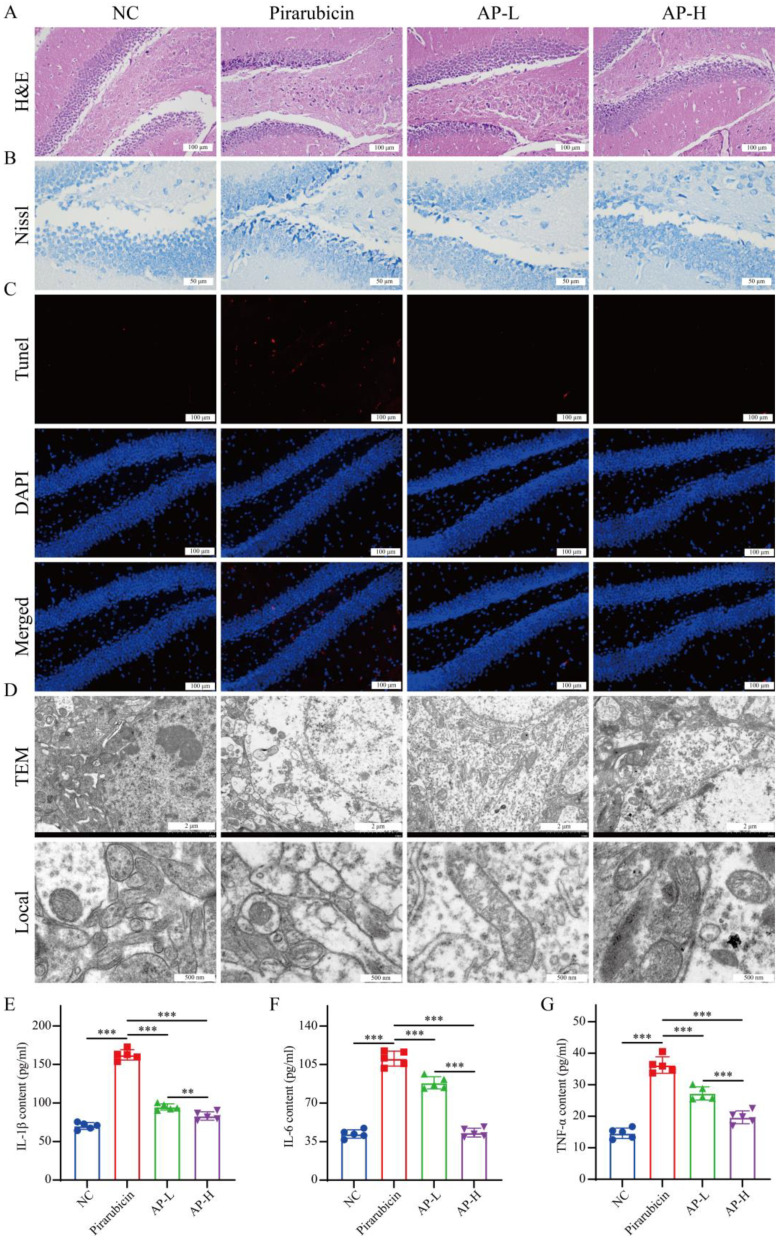
AP effectively ameliorated hippocampus damage in mice with chemotherapy. (**A**) H&E staining. (**B**) Nissl staining. (**C**) TUNEL assay. (**D**) TEM. (**E**–**G**) Levels of inflammatory factors involving IL-1β, IL-6, and TNF-α in hippocampus. Data are presented as mean ± SD; comparison between the two groups: ** *p* < 0.01, *** *p* < 0.001; *n* = 5.

As the hippocampus constitutes a key regulator in depression modulation [5,13], we investigated its involvement in AP-mediated amelioration of depressive-like behaviors. In this study, H&E staining, Nissl staining, TUNEL assay, TEM, and inflammatory factors of the hippocampus were detected. As shown in Figure 3A, H&E staining demonstrated preserved hippocampal cytoarchitecture with distinct nuclei and cytoplasm in the NC group, whereas the pirarubicin group exhibited severe structural disorganization; this pathology was mitigated in AP-L and restored to near-baseline levels in AP-H. As shown in Figure 3B, the pirarubicin group showed severe degenerative changes in neurons as the morphology of shrunken cytoplasm and condensed staining compared with NC group in the hippocampus, whereas those neural degeneration signs were alleviated in the AP-L and AP-H groups. As shown in Figure 3C, TUNEL assays showed significantly elevated apoptotic cells following pirarubicin exposure, which were attenuated in both AP treatment groups. As shown in Figure 3D, TEM confirmed ultrastructural preservation of hippocampal cell–cell organelles in AP-treated mice, contrasting with disrupted mitochondrial and cellular integrity in pirarubicin specimens. Pro-inflammatory cytokine levels (IL-1β, IL-6, and TNF-α) in the hippocampus were measured via ELISA to assess brain inflammation in mice. The results indicate that IL-1β, IL-6, and TNF-α levels were significantly elevated in the hippocampus of the pirarubicin group compared to the NC group. Compared with the pirarubicin group mice, the levels of IL-1β, IL-6, and TNF-α in the hippocampus of mice in the AP-L and AP-H groups significantly decreased (Figure 3E–G).

### 2.4. AP Strengthens Intestinal Barrier

**Figure 4:**
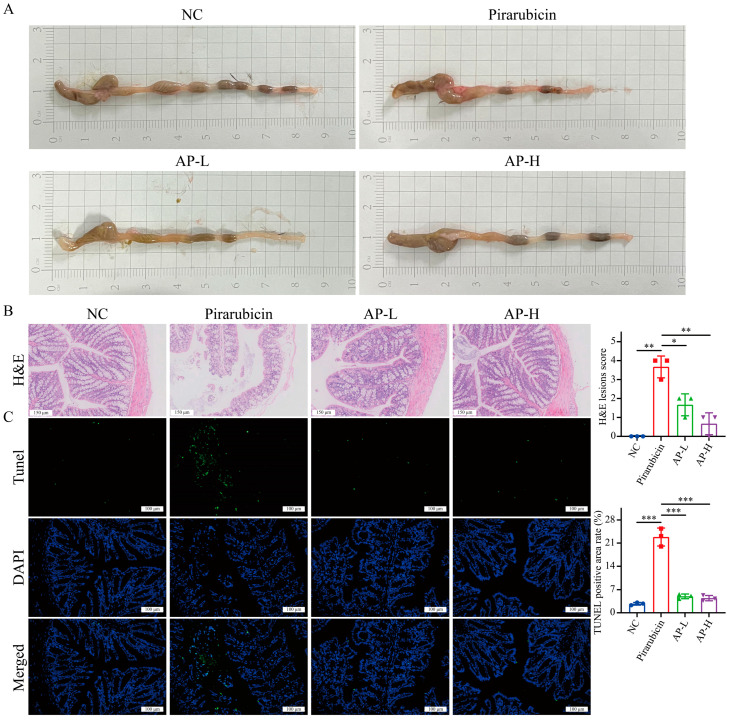
AP reduces chemotherapy-induced colonic damage. (**A**) White light map of mice intestine. (**B**) H&E staining of colon. (**C**) TUNEL assays of colon. Data are presented as mean ± SD; comparison between the two groups: * *p* < 0.05, ** *p* < 0.01, *** *p* < 0.001; *n* = 3.

Given that gut barrier dysfunction may mediate neurotoxicity and neuroinflammation through the gut–brain axis [13], we investigated whether AP’s ameliorative effects on hippocampal neuronal damage involve mechanisms of intestinal restoration. Compared with the NC group, the intestinal walls of the pirarubicin group showed obvious redness, swollen bleeding spots, and thinning. AP treatment can significantly improve macroscopic damage to the intestine caused by pirarubicin (Figure 4A). H&E staining revealed that the colon tissue of the NC group remained intact, with orderly epithelial cell arrangement and no signs of congestion or edema. In contrast, the colons of pirarubicin-treated mice exhibited epithelial cell necrosis, disordered crypt structure, and even tissue loss, with substantial infiltration of inflammatory cells in the mucosa and submucosa. However, in the AP-L and AP-H groups, colon tissue damage was significantly reduced, and there was less inflammatory cell infiltration compared to the pirarubicin group (Figure 4B). TUNEL assays showed significantly elevated intestinal apoptotic cells following pirarubicin exposure, which were attenuated in both AP treatment groups (Figure 4C).

**Figure 5:**
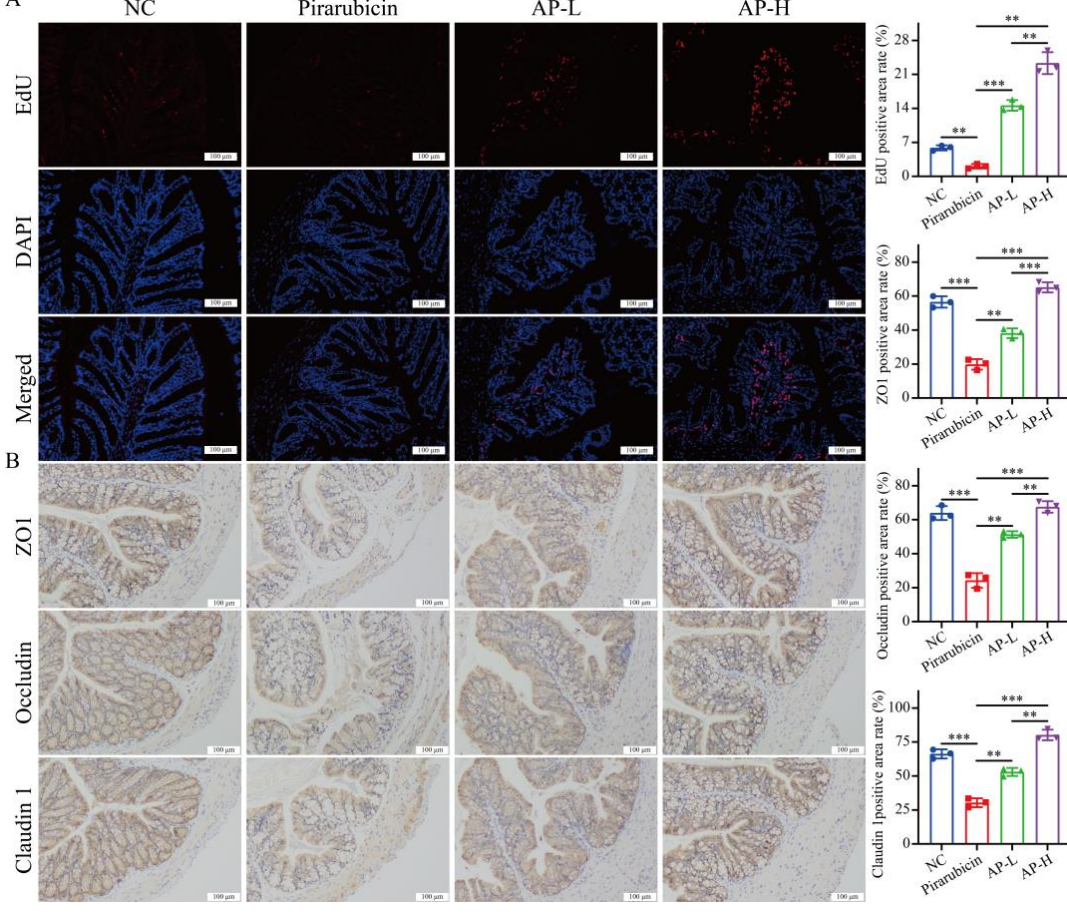
AP enhances the repair of the intestinal barrier. (**A**) EdU labeling of colon. (**B**) IHC detection of ZO1, occludin, and claudin 1 in colon. Data are presented as mean ± SD; comparison between the two groups: ** *p* < 0.01, *** *p* < 0.001; *n* = 3.

Furthermore, the repair and tight junction of the intestinal epithelium is another key aspect of intestinal barrier function. EdU labeling staining results show that AP treatment significantly promoted intestinal cell proliferation in a dose-dependent manner (Figure 5A). IHC staining results show that pirarubicin treatment significantly reduced the expression of ZO1, occludin, and claudin 1 in colon. However, treatment with AP significantly enhanced the expression of ZO1, occludin, and claudin 1 in colon (Figure 5B).

### 2.5. AP Restores Community Structure of Intestinal Flora

#### 2.5.1. Diversity Analysis

**Figure 6:**
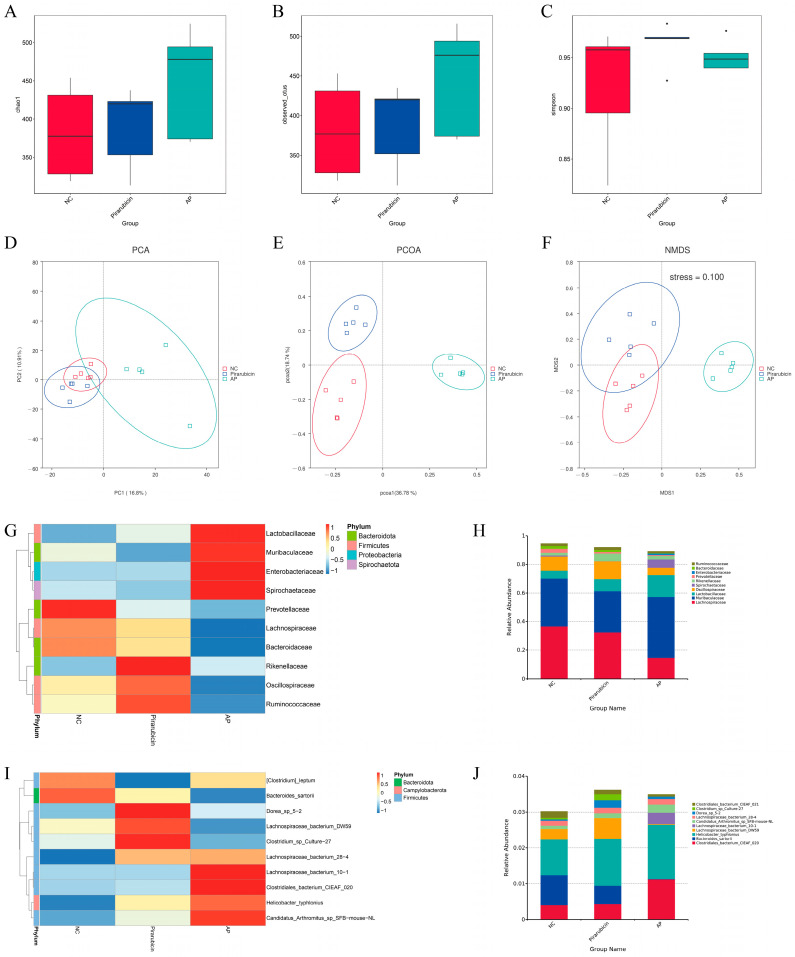
AP modulates gut microbial diversity and composition in chemotherapy-treated mice. (**A**–**C**) Box plots of alpha diversity indices, including Chao1, Observed_otus, and Simpson. (**D**–**F**) Beta diversity was analyzed using PCA, PCoA, and NMDS methods. (**G**,**H**) Heatmap and stacked bar chart showing the relative abundance of the top 10 bacterial families. (**I**,**J**) Graphical representation of the relative abundance of the top 10 bacterial species at the species level, presented as a heatmap and a stacked bar chart (*n* = 5).

It is well known that the gut microbiota plays an important role in maintaining intestinal digestion and absorption, regulating intestinal barrier function, and even modulating intestinal immunity [12]. In this study, to investigate the composition of the gut microbiota, we conducted 16S rDNA sequencing on cecal contents to analyze the gut microbiota profiles of the three mouse groups. Alpha diversity analysis includes the Chao1 index, Observed_otus index, and Simpson index. The Chao1 index is an estimator of species richness within microbial communities, while the Observed_otus index serves as a key indicator for describing microbial community diversity. Compared to the other two groups, the AP group exhibited an increasing trend in both the Chao1 index and Observed_otus index (Figure 6A,B). In addition, the Simpson index, also known as the dominance index, measures the concentration of dominance. As an inverse measure of diversity, a higher Simpson index indicates greater unevenness in species abundance distribution within the community. The analysis revealed that the Simpson indices in the pirarubicin group were significantly higher than those in the NC group. Interestingly, the indices of the AP groups exhibited a trend towards normalization compared to the pirarubicin group (Figure 6C).

Beta diversity was evaluated using Principal Component Analysis (PCA), Principal Coordinate Analysis (PCoA), and Non-metric Multidimensional Scaling (NMDS) to examine structural differences in the gut microbiota among the different intervention groups. Both PCA and PCoA results show significant differences between the NC and pirarubicin groups, with a notable alteration in the intestinal flora structure following AP treatment compared to the pirarubicin group (Figure 6D,E). Additionally, the NMDS analysis of beta diversity supported the patterns observed in both PCA and PCoA, with a stress value of 0.1 (below the 0.2 threshold), confirming the robustness and reliability of the observed results (Figure 6F).

#### 2.5.2. Species Analysis, LefSe Analysis, and Functional Predictive Analysis

**Figure 7:**
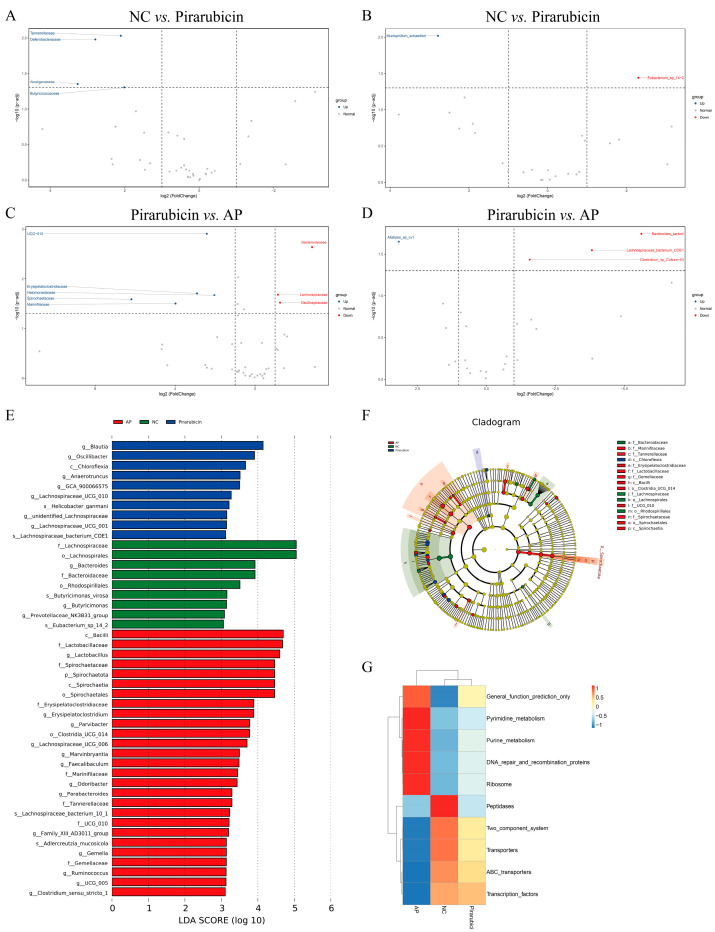
Gut microbiota changes in different groups. (**A**,**B**) Volcano plots depicting differential microbial features between the NC and pirarubicin groups at the family and species levels, respectively. (**C**,**D**) Volcano plots depicting differential microbial features between the pirarubicin and AP groups at the family and species levels, respectively. (**E**) Cladograms generated via LEfSe illustrate the differences in bacterial taxa among the NC, pirarubicin, and AP groups. (**F**) Taxonomic cladogram from LEfSe analysis. (**G**) PICRUSt functional abundance clustering heatmap. *n* = 5.

The impact of different treatment regimens on the relative abundance of gut microbiota was further investigated. At the family level, the relative abundance of *Lactobacillaceae*, *Muribaculaceae*, *Enterobacteriaceae*, *Spirochaetaceae*, *Prevotellaceae*, *Lachnospiraceae*, *Bacteroidaceae*, *Rikenellaceae*, *Oscillospiraceae*, and *Ruminococcaceae* changed significantly after different treatment (Figure 6G,H). In addition, chemotherapy led to a significant increase in the relative abundance of *Tannerellaceae*, *Deferribacteraceae*, *Alcaligenaceae*, and *Butyricicoccaceae* (Figure 7A). After the AP intervention, the relative abundance of *UCG-010*, *Erysipelatoclostridiaceae*, *Halomonadaceae*, *Spirochaetaceae*, and *Marinifilaceae* increased, while the relative abundance of *Bacteroidaceae*, *Lachnospiraceae*, and *Oscillospiraceae* decreased prominently (Figure 7C). At the species level, the relative abundance of *[Clostridium] leptum*, *Bacteroides sartorii*, *Dorea* sp. *5-2*, *Lachnospiraceae bacterium DW59*, *Clostridium* sp. *Culture-27*, *Lachnospiraceae bacterium 28-4*, *Lachnospiraceae bacterium 10-1*, *Clostridiales bacterium CIEAF 020*, *Helicobacter typhlonius*, and *Candidatus Arthromitus* sp. *SFB-mouse-NL* changed significantly after different treatment (Figure 6I,J). In addition, chemotherapy led to an increase in the relative abundance of *Mucispirillum schaedleri*, while the relative abundance of *Eubacterium* sp. *14-2* decreased significantly (Figure 7B). After the AP intervention, the relative abundance of Alistipes sp. cv1 increased, while the relative abundance of *Bacteroides sartorii, Lachnospiraceae bacterium COE1*, and *Clostridium* sp. *Culture-54* decreased prominently (Figure 7D).

Linear discriminant analysis (LDA) Effect Size (LEfSe) was used to identify biomarkers with statistically significant differences between groups. The LDA score histogram revealed 46 biomarkers with significant inter-group differences (*p* < 0.05, LDA > 3.0) (Figure 7E,F). Among these, 10 differentially abundant clades were found in the pirarubicin group, and 27 in the AP group (*p* < 0.05, LDA > 3.0) (Figure 7E,F). The results of PICRUSt functional abundance clustering showed significant differences in general function prediction only, pyrimidine metabolism, purine metabolism, DNA repair and recombination proteins, ribosome, peptidases, two_component system, transporters, ABC transporters, and transcription factors among different groups (Figure 7G).

### 2.6. AP Improves Microbial Metabolites

**Figure 8:**
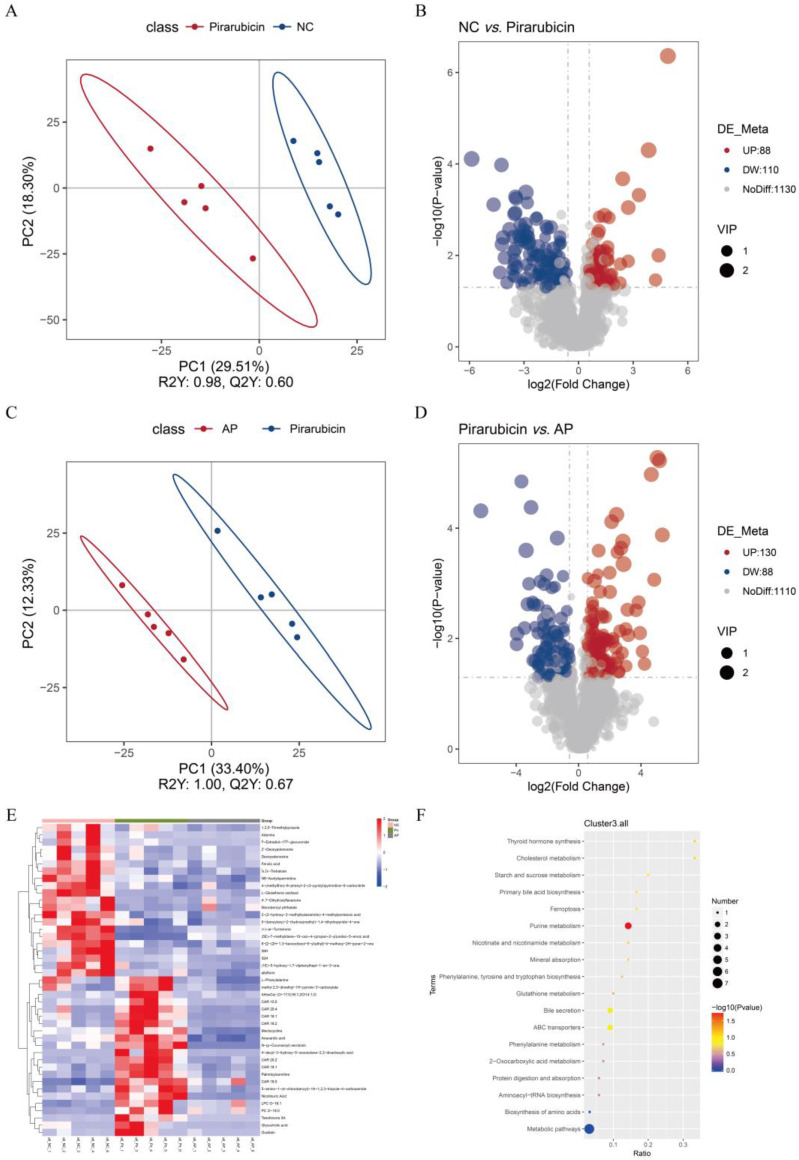
AP modulates the intestinal metabolites in chemotherapy-treated mice. (**A**) PLSDA-score plots for discriminating the fecal metabolome between the NC and pirarubicin groups. (**B**) Volcano plot of fecal metabolomics comparing the NC and pirarubicin groups in mice. (**C**) PLSDA-score plots for discriminating the fecal metabolome between the pirarubicin and AP groups. (**D**) Volcano plot of fecal metabolomics comparing the pirarubicin and AP groups in mice. (**E**) Heatmap of differential metabolites between groups. (**F**) Enrichment analysis of KEGG pathways for inter-group differential metabolites. *n* = 5.

To further investigate changes in intestinal metabolites in chemotherapy-treated mice and the regulatory effects of AP, untargeted metabolomics was used to analyze metabolite profiles in mouse fecal samples, and statistically significant differential metabolites were identified. PLS-DA was performed on the samples to assess the overall metabolic differences and variability among them. The results reveal a separation trend between the fecal microbiota across different metabolic groups, with no significant differences observed within the groups themselves (Figure 8A,C). The results of this study are presented as a volcano plot to visually depict differential metabolites, with the horizontal axis representing the log2 fold change (log2FC) in relative metabolite content between groups. A larger absolute value indicates a greater difference between groups. In comparison to the NC group, 88 metabolites were significantly increased, while 110 metabolites were decreased in the pirarubicin group (Figure 8B). In the AP group, 130 metabolites were significantly increased, while 88 metabolites were decreased compared to the pirarubicin group (Figure 8D). The heatmap reveals 43 metabolites with significant differences between groups (Figure 8E). Differential metabolites were annotated and enriched using the Kyoto Encyclopedia of Genes and Genomes (KEGG) database. The analysis revealed significant inter-group differences in pathways related to ferroptosis, mineral absorption, glutathione metabolism, ABC transporters, and various other enzymes and signaling pathways (Figure 8F).

### 2.7. AP Reduces Ferroptosis in Intestinal Tissue

**Figure 9:**
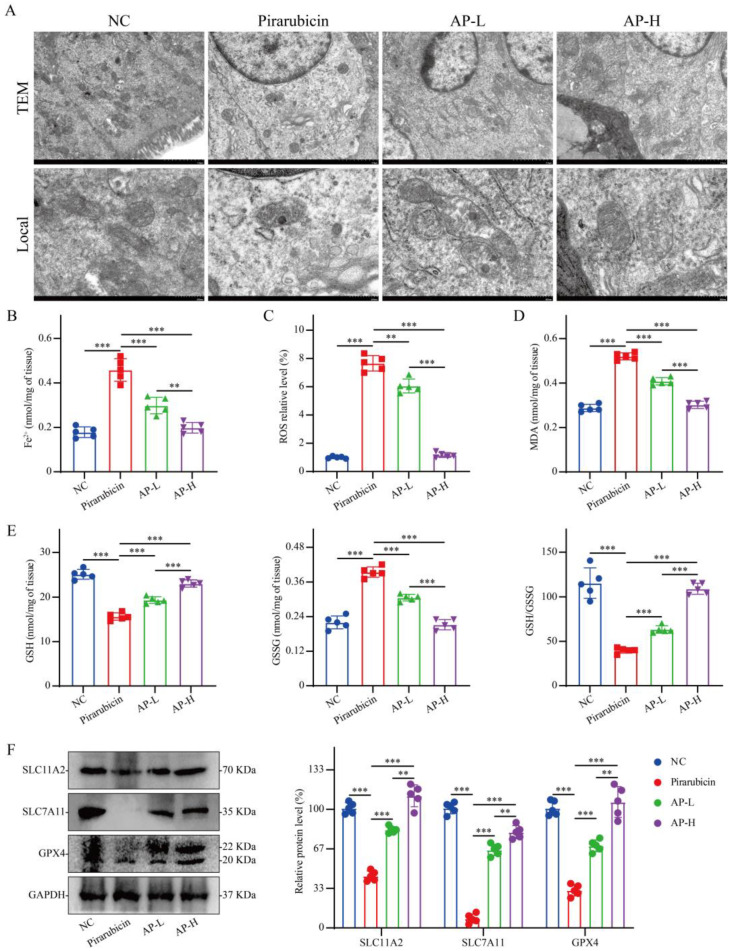
AP protects intestinal tissues from ferroptosis induced by pirarubicin. (**A**) TEM; scale bar 2 μm or 500 nm. (**B**–**E**) The levels of Fe^2+^, ROS, MDA, GSH, and GSSG in the colon tissue. (**F**) The protein expression level and relative quantification of SLC11A2, SLC7A11, and GPX4 in colon tissue. Data are presented as mean ± SD; comparison between the two groups: ** *p* < 0.01, *** *p* < 0.001; *n* = 5.

Based on the results of the enrichment analysis, we further investigated the relationship between ferroptosis in the mouse intestine and the therapeutic effect of AP. TEM revealed that mitochondria in the pirarubicin group exhibited severe ultrastructural damage, characterized by markedly reduced volume, increased double-layer membrane density, loss or fragmentation of cristae, and evident shrinkage and rupture of the outer membrane. In contrast, the mitochondrial morphology in the AP-treated groups more closely resembled that of the NC group, with the AP-H group showing the most prominent protective effects (Figure 9A). In intestinal tissue, pirarubicin treatment increased the levels of Fe^2+^, ROS, MDA, and GSSG while decreasing GSH, compared to the NC group. Both low and high doses of AP (AP-L and AP-H) significantly reversed these effects, reducing the concentrations of Fe^2+^, ROS, MDA, and GSSG and elevating GSH content relative to the pirarubicin group (Figure 9B–E). To further confirm the increased levels of ferroptosis in the intestinal tissue from the pirarubicin group, we extracted proteins from the intestinal tissue for analysis. Western blot analysis of the intestinal tissue indicated that the SCL11A2, SLC7A11, and GPX4 protein expression levels were decreased compared with those in the NC group, while AP could increase the expression of these proteins in a dose-dependent manner (Figure 9F).

### 2.8. Intestinal Flora Mediates the Anti-Depressant Effect of AP

**Figure 10:**
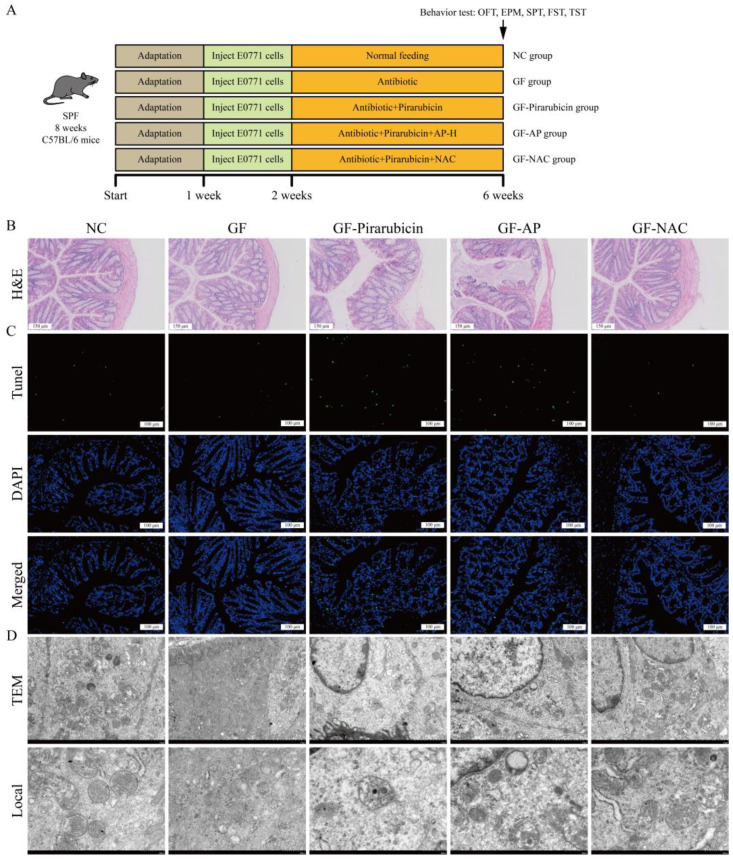
The reduction in intestinal flora compromised the protective effects of AP on the gut. (**A**) Schematic showing the experimental design and timeline. (**B**) H&E staining of colon. (**C**) TUNEL assays of colon. (**D**) TEM; scale bar 2 μm or 500 nm.

In this study, we observed that AP alleviated depression-like behaviors in pirarubicin-treated mice by regulating gut microbiota and microbial metabolites. To further validate that modulation of the gut microbiota plays a key role in AP’s effect on depression-like behaviors, we examined the behavioral consequences of changes in the intestinal microbiota. This was achieved using pseudo-germ-free mice and further confirmed with the ferroptosis inhibitor NAC, elucidating AP’s underlying mechanism (Figure 10A). As shown in Figure 10B–D, depletion of the gut microbiota markedly attenuated the protective effect of AP against chemotherapy-induced intestinal injury. Nevertheless, NAC still demonstrated protective efficacy following gut microbiota depletion. This suggests that AP likely requires the gut microbiota to exert its NAC-like protective effects.

**Figure 11:**
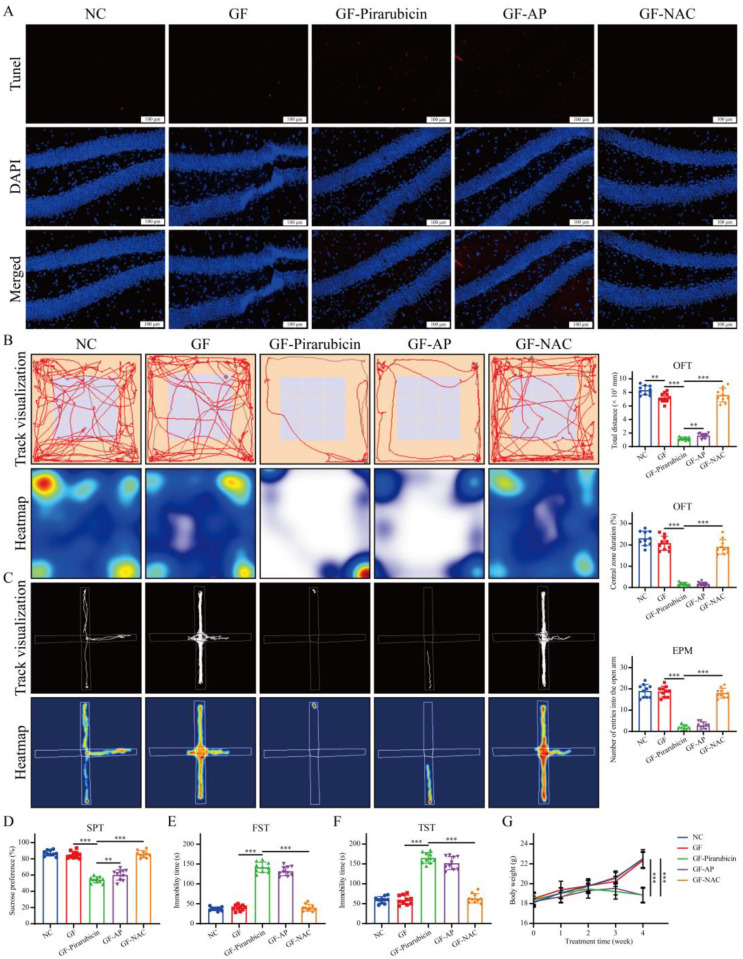
The reduction in intestinal flora compromised the antidepressant efficacy of AP. (**A**) TUNEL assay. (**B**) Track map, heatmap, total distance, and central zone duration in OFT. (**C**) Track map, heatmap, and number of entries into the open arm in EPM. (**D**) Sucrose preference test (SPT). (**E**) Immobility time in FST. (**F**) Immobility time in TST. (**G**) Body weight was measured every week. Data are presented as mean ± SD; comparison between the two groups: ** *p* < 0.01, *** *p* < 0.001; *n* = 10.

Additionally, consistent with intestinal pathology, hippocampal analyses revealed analogous alterations. Following microbiota depletion, AP failed to mitigate chemotherapy-induced hippocampal neuronal damage, whereas NAC substantially protected neurons against such injury (Figure 11A). As shown in Figure 11B–F, following gut microbiota depletion, the efficacy of AP in alleviating CID was significantly attenuated. In contrast, NAC maintained robust behavioral improvements even after gut microbiota ablation. In pseudo-germ-free mice, pirarubicin treatment induced significant body weight loss compared to the GF group, which was not reversible with AP. However, NAC administration substantially restored pirarubicin-induced weight reduction (Figure 11G).

## 3. Discussion

Recent research has demonstrated that herbs and their natural compounds hold significant potential in alleviating neuroinflammation, anxiety, and depression associated with chemotherapy [4,5]. It has been discovered that AP can affect the fermentation function of the human gut microbiota [25], but whether this effect is related to intestinal repair and the regulation of depressive-like behaviors remains unknown. In the present study, we demonstrated that AP alleviates depression-like behaviors in chemotherapy-treated mice, while also suppressing hippocampal inflammation and neuronal injury and restoring intestinal barrier function. Specifically, AP rebalanced the gut microbiota and alleviated the disruption of intestinal microbial metabolites. It is worth noting that in the pseudo-germ-free mouse model, the effect of AP in improving chemotherapy-induced depressive-like behavior significantly decreased, indicating that the antidepressant effects of AP in chemotherapy are primarily driven by the modulation of the intestinal microenvironment.

CID is a common complication, affecting approximately 45% of patients [26]. While it is widely acknowledged that CID significantly impacts patients’ quality of life and rehabilitation, its underlying pathophysiology remains poorly understood, hindering the development of effective clinical interventions. Recently, natural product therapies, such as polysaccharides, have gained increasing attention for treating CID due to their remarkable clinical effectiveness and minimal side effects [27]. We previously confirmed that the traditional Chinese medicine formula has a therapeutic effect on CID [5,28]. Based on the principles of traditional Chinese medicine, tonifying polysaccharides serve as the primary active constituents in Chinese herbal medicine responsible for exerting immunomodulatory effects and assisting the organism in counteracting adverse damage [29]. *Atractylodes macrocephala* Koidz., as a traditional tonifying medicinal herb, has been analyzed to confirm that AP can prevent tissue damage caused by drugs [30]. Further research has revealed that AP possesses anti-inflammatory biological activity and can inhibit the M1 type polarization of macrophages [31]. However, whether AP has a therapeutic effect on CID has not been reported until this study, which demonstrated that AP does have a therapeutic effect on CID and further revealed its potential mechanism in treating CID.

The relationship between gut microbiota dysbiosis and pathological changes in the central nervous system has been a hot topic in recent research. Increasing evidence suggests the existence of a “gut–brain axis” between the gut microbiota and the brain, which plays a crucial role in central nervous system functions and behavioral responses. Dysbiosis of the gut microbiota is a recognized contributor to neuroinflammation and cognitive impairment [32]. Furthermore, its role in the pathogenesis of depression has been widely investigated. Studies have shown that the gut microbiota can regulate neurotransmission, neuroinflammation, and behavior through the vagus nerve and the production of microbial metabolites and immune mediators [33]. Although understanding is still limited regarding the use of gut microbiota modulation to treat diseases, animal studies have shown its potential in the treatment of depression [33]. Dysbiosis of the gut microbiota is closely associated with pathological changes in various central nervous system disorders. A deeper understanding of how the gut microbiota interacts with the central nervous system could provide new strategies for preventing and treating CID [34]. Although anthracycline drugs have poor blood–brain barrier penetration and cannot directly enter brain tissue to damage the nervous system, their side effects remain unavoidable during treatment [4], especially as pirarubicin has been reported to have significant adverse effects on the intestinal barrier, leading to dysbiosis and a decline in intestinal barrier defense function [27]. This process will cause a large amount of inflammatory substances to enter the circulatory system, ultimately leading to damage and dysfunction of the central nervous system tissues and resulting in depressive-like behaviors [35].

Studies have confirmed that *Bacteroidaceae* [36], *Lachnospiraceae* [37], and *Oscillospiraceae* [38] can significantly promote depressive-like behaviors, whereas Alistipes has been proven to alleviate depressive symptoms because it promotes the production of short-chain fatty acids, which are a type of fatty acid with antidepressant effects [39,40]. Furthermore, the homeostasis of *Clostridium* is associated with the regulation of depression and has potential antidepressant effects [39]. Interestingly, we found that AP can regulate the gut microbiota in chemotherapy-treated mice, including *Bacteroidaceae*, *Lachnospiraceae*, *Oscillospiraceae*, *Alistipes*, and *Clostridium*. Some other studies have shown that *Alistipes* may influence the metabolism of glycocholic acid, L-phenylalanine, and palmitoylcarnitine [41,42]. Elevated phenylalanine levels have been confirmed to cause cognitive impairment and brain abnormalities, potentially by reducing BDNF mRNA and protein expression, thereby diminishing neurite development and promoting neuronal death [43,44]. However, after gut microbiota depletion, the neuroprotective and antidepressant effects of AP were significantly weakened. These findings suggest that AP treats CID by regulating the gut microbiota and its metabolites.

In addition, inflammation infiltration is an important factor in neuronal damage [5]. We found that pro-inflammatory cytokine levels were significantly elevated in the hippocampal tissue of chemotherapy-treated mice, accompanied by neuronal damage. The hippocampus is a critical neural center for emotional regulation, so inflammation-induced neuronal damage in the hippocampus may be an important mechanism of CID [13,27]. The intestinal microecological environment is one of the main links in regulating the inflammatory level of the body, and damage to the intestinal barrier is a major factor in the leakage of inflammatory substances from the gut [27]. Anthracycline chemotherapy drugs, as non-targeted tumor treatments, can easily damage normal tissues. Numerous studies have shown that anthracycline chemotherapy drugs induce mitochondrial dysfunction by directly binding to cardiolipin on the mitochondrial inner membrane, leading to the production of large amounts of ROS within cells [45,46]. This, in turn, causes ferroptosis in normal cells, ultimately resulting in tissue damage [47]. Our results show that during chemotherapy, the gut of mice exhibited significant characteristics of ferroptosis, which may be an important pathological mechanism of chemotherapy-induced intestinal barrier disruption. The intestinal barrier and gut microbiota are mutually complementary. Damage to the intestinal barrier leads to gut microbiota dysbiosis. Therefore, restoring the normal state of the gut microbiota may be an effective approach to repairing the intestinal barrier [12]. AP downregulated the abundance of *Bacteroidaceae*, *Lachnospiraceae*, *Oscillospiraceae*, and *Clostridium*. *Bacteroidaceae* is believed to induce ferroptosis in intestinal tissues and disrupt the stability of the intestinal barrier [48], and *Clostridium* may trigger ferroptosis in the intestinal tissues and increase inflammatory infiltration [49]. Furthermore, L-phenylalanine, a major product of *Bacteroidaceae* and *Oscillospiraceae* [50], has been reported to potentially trigger tissue inflammation and ferroptosis. AP reduces the content of L-phenylalanine in intestinal metabolites by interfering with the intestinal microbiota, which may be a major reason for AP alleviating chemotherapy-induced ferroptosis in intestinal tissue. The important point is that after the intestinal microbiota was cleared, the protective effect of AP on the intestine almost vanished. These results suggest that AP may alleviate chemotherapy-induced intestinal barrier disruption caused by ferroptosis in intestinal tissue by modulating the gut microbiota and its metabolites.

**Figure 12:**
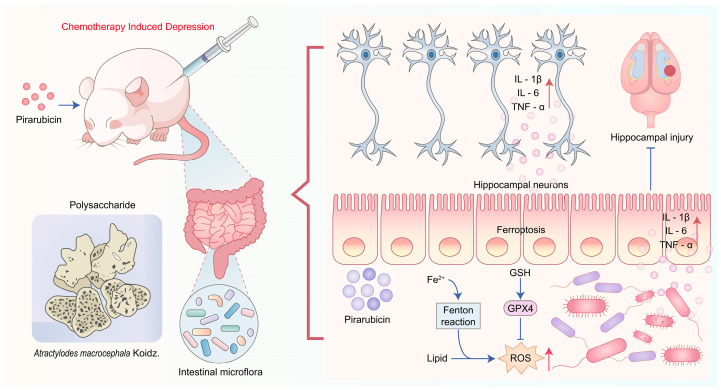
Potential mechanisms of AP in breast cancer chemotherapy mice. AP alleviates CID by regulating the gut–brain axis.

Overall, we believe that the side effects of chemotherapy cause ferroptosis in intestinal tissue, disrupting the stability of the intestinal barrier. This pathological change allows pro-inflammatory substances to enter the circulatory system, ultimately leading to hippocampal neuronal damage and functional abnormalities, thereby mediating the onset of depression. AP influences the gut microbiota, promotes intestinal barrier repair, and intervenes in depression-related bacteria and their metabolites, effectively treating CID (Figure 12). Although our study confirmed that AP can treat CID, it did not further validate the specific bacteria involved. In addition, in the omics analysis, the AP-L group and the pseudo-germ-free mice were not included. In future studies, we can conduct fecal microbiota transplantation (FMT), and then conduct functional validation targeting particular bacterial species to further elucidate the key bacteria responsible for AP’s therapeutic effects on CID, thereby providing a crucial theoretical foundation for developing gut microbiota-targeted interventions for the clinical treatment of CID.

## 4. Materials and Methods

### 4.1. AP Preparation

*Atractylodes macrocephala* Koidz. (Anhui, China) was supplied and characterized by the Guangdong Provincial Hospital of Chinese Medicine (Guangzhou, Guangdong, China). *Atractylodes macrocephala* Koidz. was soaked in water at a ratio of 1:10 and boiled for 1.5 h twice. The supernatant was collected, filtered, and concentrated. Subsequently, the polysaccharide was precipitated from the clear solution with ethanol at 4 °C for 24 h. Then, the AP sample was freeze-dried to acquire a fine powder, which was stored at −80 °C. Furthermore, the structural and physicochemical characteristics of AP were characterized through monosaccharide composition analysis, molecular weight determination, Fourier-transform infrared spectroscopy (FT-IR) analysis, and scanning electron microscopy (SEM) [51].

### 4.2. Other Chemicals and Reagents

Dulbecco’s modified Eagle’s medium with high glucose, L-glutamine, and phenol red (DMEM; Cat. #11965092), fetal bovine serum (FBS; Cat. #10270106), penicillin/streptomycin (Cat. #10378016), phosphate-buffered saline (PBS, Cat. #10010023), and trypsin-EDTA (Cat. #25200072) were supplied by Gibco (Carlsbad, CA, USA). Pirarubicin (Cat. #HY-13725) was purchased from MedChemExpress (Shanghai, China). Nissl Staining Solution (Cat. #C0117), a One Step TUNEL Apoptosis Assay Kit (Cat. #C1086 and C1089), a BeyoClick™ EdU Cell Proliferation Kit with AF555 (Cat. #C0075S), a mouse IL-1β ELISA Kit (Cat. #PI301), a mouse IL-6 ELISA Kit (Cat. #PI326), a mouse TNF-α ELISA Kit (Cat. #PT512), a Lipid Peroxidation MDA Assay Kit (Cat. #S0131S), and a GSH and GSSG Assay Kit (Cat. #S0053) were supplied by the Beyotime Institute of Biotechnology (Shanghai, China). Primary antibodies anti-ZO1 (Cat. #ab276131), anti-Occludin (Cat. #ab216327), anti-Claudin 1 (Cat. #ab211737), anti-DMT1 (SLC11A2, Cat. #ab262715), anti-xCT (SLC7A11, Cat. #ab307601), anti-Glutathione Peroxidase 4 (GPX4, Cat. #ab125066), and anti-GAPDH (Cat. #ab8245) were supplied by Abcam Plc (Cambridge, UK). The Iron Assay Kit (Sigma-Aldrich, Cat. #MAK025, St. Louis, MO, USA) and the Tissue ROS Assay Kit (Bestbio, Cat. #BB-470532, Shanghai, China) were used for the respective measurements.

### 4.3. Cell Culture

E0771 cells (ATCC, RRID:CVCL_GR23), derived from spontaneous mammary tumor in C57BL/6 mouse, were maintained in high-glucose DMEM supplemented with 10% fetal bovine serum (FBS), 100 U/mL penicillin, and 100 µg/mL streptomycin. Cells were cultured at 37 °C in a humidified tri-gas incubator with 5% CO_2_, with medium replenished every 48 h. Upon reaching 90% confluence, cells were routinely passaged, and those in the logarithmic growth phase were selected for subsequent experiments.

### 4.4. Establishment, Grouping, and Administration of Animal Models

Ninety specific pathogen-free (SPF)-grade female C57BL/6 mice (8 weeks old, body weight 19 ± 1 g) were purchased from Guangdong Charles River Laboratory Animal Technology Co., Ltd. (Guangzhou, Guangdong, China) and housed at the Center for Laboratory Animal Management, Jinan University. All mice were established with breast cancer models following standardized procedures [52].

Experiment 1: Mice were randomly assigned to one of four groups (Figure 2A): NC group (*n* = 10); pirarubicin group (*n* = 10), which were administered pirarubicin (5 mg/kg/wk, i.p.); AP-L group (*n* = 10), which were treated with pirarubicin and given a low dose of AP (0.1 g/kg/d, orally); AP-H group (*n* = 10), which were treated with pirarubicin and received a high dose of AP (0.3 g/kg/d, orally).

Experiment 2: Mice were randomly assigned to one of five experimental groups (Figure 10A): NC group (*n* = 10): normal control; GF group (*n* = 10): mice received antibiotic water ad libitum (1 mg/mL ampicillin, 1 mg/mL metronidazole, 1 mg/mL neomycin, and 0.5 mg/mL vancomycin); GF-pirarubicin group (*n* = 10): mice received antibiotic water and pirarubicin treatment; GF-AP group (*n* = 10): mice received antibiotic water, pirarubicin, and high-dose AP; GF-NAC group (*n* = 10): mice received antibiotic water supplemented with 0.5% NAC, along with pirarubicin.

Body weight and tumor volume were measured weekly. Tumor volume was calculated using the formula V = (length × width^2^)/2. The experimental protocols were reviewed and approved by the Ethics Committee for Laboratory Animal Welfare of Jinan University, in compliance with the principles of animal protection, welfare, and ethics. The approval number for animal ethics is IACUC-20240318-02.

### 4.5. Behavioral Analysis

Depressive-like behaviors in all mouse groups were assessed using established behavioral paradigms, according to published protocols: the open field test (OFT), elevated plus maze (EPM), and sucrose preference test (SPT) [13,53].

### 4.6. Euthanasia and Sample Collection

Mice were administered 5 mg/kg EdU via intraperitoneal injection daily for three consecutive days before euthanasia. Upon conclusion of the drug treatment and behavioral tests, the animals were euthanized with an overdose of sodium pentobarbital (100 mg/kg, i.p.). Colon tissue, cecal content, and brain tissue were rapidly harvested and appropriately stored for subsequent analysis.

### 4.7. Pathological Analysis

Fresh colon and brain tissue samples were fixed in 4% paraformaldehyde for 48 h. Subsequently, the tissues underwent processing, dehydration, and paraffin embedding. Sections were prepared at a thickness of 4 μm using a microtome from the paraffin-embedded tissue blocks. Subsequently, histological sections were subjected to hematoxylin and eosin (H&E) staining, Nissl staining, TUNEL assay, EdU labeling, and immunohistochemistry (IHC) using well-established protocols [4,52,54]. Structural alterations in staining were examined under a fluorescence microscope within comparable regions of the tissue. Semiquantitative scoring of tissue lesions was calculated according to Wu et al. [28]. Briefly, lesions in 3 fields were chosen randomly from each slide for each mouse and averaged. The lesions were scored in a blinded manner (score scale: 0 = normal; 1 ≤ 25%; 2 = 26–50%; 3 = 51–75%; and 4 = 76–100%).

Fresh colon tissue and brain tissue were fixed in 2.5% glutaraldehyde for 4 h. Transmission electron microscope (TEM) specimens were processed according to established ultramicrotomy protocols [55] and observed under a TEM for the ultrastructural details of the tissues.

### 4.8. Enzyme-Linked Immunosorbent Assay (ELISA)

An IL-1β ELISA kit, IL-6 ELISA kit, and TNF-α ELISA kit were used to measure inflammatory factor concentrations in hippocampal tissues. In summary, fresh tissue samples were collected and analyzed using a microplate reader in accordance with the manufacturer’s kit protocols. All experiments were conducted in five independent replicates, and results are reported in pg/mL.

### 4.9. 16S rDNA Sequencing

Cecal contents underwent 16S rDNA sequencing according to established methodologies [56]. Genomic services including library preparation and sequencing were performed by Novogene Co., Ltd. (Beijing, China), with subsequent bioinformatic analyses conducted through their proprietary cloud platform. In addition, owing to financial limitations, only the following groups were included in the analysis: the NC group, the pirarubicin group, and the pirarubicin group receiving high-dose AP (AP group).

### 4.10. Metabolomics Analysis

Cecal contents were subjected to metabolomic profiling following established protocols [56]. This experiment was contracted to Novogene Co., Ltd. for execution, with subsequent bioinformatic analyses performed on the company’s cloud platform. Similarly, this experiment also only analyzed three groups.

### 4.11. Ferroptosis-Related Index Detection

An iron assay kit, tissue ROS assay kit, lipid peroxidation MDA assay kit, and GSH and GSSG assay kits were used to measure ferroptosis-related index concentrations. In summary, fresh tissue samples were collected and analyzed using a fluorescence microplate reader following the manufacturer’s protocol provided with the assay kit. These experiments were performed in five replicates. Experimental data were presented in accordance with the manufacturer’s recommended format for the assay kit.

### 4.12. Western Blot (WB)

According to the established experimental protocol [28], SLC11A2 (1:1000), SLC7A11 (1:1000), and GPX4 (1:1000) protein expression in colon tissues was analyzed via WB. Chemiluminescence was triggered with a chemiluminescent reagent. Protein separation membranes were scanned and analyzed using an image analyzer (Bio-Rad, Hercules, CA, USA). GAPDH served as the loading control.

### 4.13. Statistical Analysis

Statistical analyses were performed using SPSS 28.0. Data that met the assumptions of normality and homogeneity of variance were analyzed using one-way analysis of variance (ANOVA), while the non-parametric Kruskal–Wallis H test was employed for data violating these assumptions. All statistical analyses were performed and visualized using GraphPad Prism version 9.0 (GraphPad Software, Boston, MA, USA). Statistically significant differences were defined as those with a *p*-value less than 0.05.

## 5. Conclusions

In summary, the development and progression of CID are closely associated with the integrity of the intestinal barrier. AP helps reduce inflammation and safeguard the intestinal mucosa by modulating intestinal bacterial metabolism, which in turn protects hippocampal neurons and alleviates neuroinflammation, ultimately aiding in the treatment of CID.

## Data Availability

The datasets used and/or analyzed during the current study are available from the corresponding author upon reasonable request. The raw 16S rDNA sequencing data generated in this study were deposited in the SRA database under accession number PRJNA1334163.

## Abbreviations

The following abbreviations are used in this manuscript:

AP	*Atractylodes macrocephala* Koidz. polysaccharide
CID	Chemotherapy-induced depression
BBB	blood–brain barrier
FT-IR	Fourier-transform infrared spectroscopy
SEM	scanning electron microscopy
DMEM	Dulbecco’s modified Eagle’s medium
FBS	fetal bovine serum
PBS	phosphate-buffered saline
TUNEL	Terminal deoxynucleotidyl transferase-mediated dUTP nick end labeling
IL-1β	Interleukin-1β
ELISA	Enzyme-linked immunosorbent assay
IL-6	Interleukin-6
TNF-α	Tumor necrosis factor-alpha
MDA	Malondialdehyde
GSH	Glutathione
GSSG	Glutathione disulfide
SPF	specific pathogen-free
OFT	open field test
EPM	elevated plus maze
SPT	sucrose preference test
H&E	hematoxylin and eosin
IHC	immunohistochemistry
TEM	transmission electron microscope
ANOVA	one-way analysis of variance
GPC-RI-MALS	Gel permeation chromatography–refractive index–multi-angle light scattering
HPLC	High-performance liquid chromatography
PCA	Principal component analysis
PCoA	Principal coordinate analysis
NMDS	Non-metric multidimensional scaling
LDA	Linear discriminant analysis
LEfSe	LDA effect size
KEGG	Kyoto Encyclopedia of Genes and Genomes
ROS	Reactive oxygen species
NAC	N-acetylcysteine
FMT	Fecal microbiota transplantation
